# Heart rate variability during mindful breathing meditation

**DOI:** 10.3389/fphys.2022.1017350

**Published:** 2023-01-23

**Authors:** Aravind Natarajan

**Affiliations:** Google LLC, San Francisco, CA, United states

**Keywords:** heart rate variability, biofeedback, mindfulness, meditation, physiology Frontiers

## Abstract

We discuss Heart Rate Variability (HRV) measured during mindful breathing meditation. We provide a pedagogical computation of two commonly used heart rate variability metrics, i.e. the root mean square of successive differences (RMSSD) and the standard deviation of RR intervals (SDRR), in terms of Fourier components. It is shown that the root mean square of successive differences preferentially weights higher frequency Fourier modes, making it unsuitable as a biosignal for mindful breathing meditation which encourages slow breathing. We propose a new metric called the autonomic balance index (ABI) which uses Respiratory Sinus Arrhythmia to quantify the fraction of heart rate variability contributed by the parasympathetic nervous system. We apply this metric to heart rate variability data collected during two different meditation techniques, and show that the autonomic balance index is significantly elevated during mindful breathing, making it a good signal for biofeedback during meditation sessions.

## Introduction

Mindfulness has shown promise as a non-pharmaceutical intervention in the management of stress, as well as a variety of other conditions [Niazi and Niazi (2011); Khoury et al. (2013); Goldberg et al. (2018); Spijkerman et al. (2016); Pickert (2014)]. Meditation and mindfulness practices have the ability to support individuals, especially during difficult times [Behan (2020); Toniolo-Barrios and Pitt (2021)]. Mindful breathing exercises have been shown to be helpful in reducing reactivity to repetitive thoughts [Feldman et al. (2010)].

Mindfulness Based Stress Reduction (MBSR) originally introduced by Jon Kabat-Zinn [Kabat-Zinn (2003)] has proved to be an evidence based technique for the improvement of health and wellbeing [Grossman et al. (2004a)]. In a metastudy of 7 controlled and randomized controlled studies which were aggregated, MBSR was shown to have a significant positive non-specific effect compared to the absence of any treatment when comparing Cohen’s *d* measures of stress [Chiesa and Serretti (2009)]. A study involving 75 participants who engaged in an 8 weeks course on MBSR showed a significant (Cohen *d* = 1.04) decrease in stress as measured by the 10-item Perceived Stress Scale [Baer et al. (2012)]. Similarly, a study involving 53 participants who attended a 10-day Vipassana meditation retreat showed reductions in overall distress 3 months following the retreat, encompassing a spectrum of psychological symptoms (Ostafin et al. (2006)). Mindfulness based treatments are also pursued in the management of chronic pain [Chiesa and Serretti (2011); Bawa et al. (2015); Veehof et al. (2016)] and insomnia [Ong and Sholtes (2010); Ong et al. (2012; 2014); Garland et al. (2016); Wang et al. (2020)]. Mindfulness/Meditation also has the power of neuroplasticity, e.g. Hölzel et al. (2011) showed changes in regional brain gray matter density after just 8 weeks of an MBSR program.

Commercially available wearable devices are increasingly popular in the United States, and many wearable devices offer mindfulness training [Balconi et al. (2017); Chiovato et al. (2021); Balconi et al. (2019); Kim et al. (2021); Hao et al. (2017); Bhayee et al. (2016)]. A notable technique for training in mindfulness meditation is the use of biofeedback signals. Biofeedback can help individuals gain awareness of physiological processes occurring within the body, and also to consciously control those processes [Ratanasiripong et al. (2010)]. A very useful biosignal is the heart rate variability (HRV) which refers to the beat-to-beat variability in heart rate. A high HRV usually indicates good health and an increased ability to adapt to stressful situations. HRV biofeedback has been applied to the management of stress [Ratanasiripong et al. (2012)], depression [Siepmann et al. (2008); Economides et al. (2020)], and asthma [Lehrer et al. (2004)]. In a meta-analytic review of HRV biofeedback, it was shown that HRV biofeedback produces improvement in a variety of physical and emotional conditions [Lehrer et al. (2020)].

HRV is one of the best non-invasive probes of the autonomic nervous system (ANS) [Shaffer and Ginsberg (2017)]. The ANS consists of two main branches: the sympathetic branch which predominates during exercise, and stressful “fight or flight” reactions, and the parasympathetic branch which predominates during quiet, resting conditions [McCorry (2007)]. The 10th cranial nerve called the vagus nerve is the main contributor of the parasympathetic nervous system (PNS) and the provides the main parasympathetic supply to the heart [Breit et al. (2018); Bonaz et al. (2017)]. A valuable metric of vagal or parasympathetic activity is Respiratory Sinus Arrhythmia (RSA) [Berntson et al. (1993); Grossman et al. (2004b); Hayano et al. (1996); Berntson et al. (1997); Eckberg (1983); Grossman and Taylor (2007); Porges (1992); Lehrer (2022)] which is the rhythmic modulation of the heart rate in response to respiration. The heart rate increases during inhalation, and decreases during exhalation, and this phenomenon has been associated with the efficiency of pulmonary gas exchange [Hirsch and Bishop (1981); Hayano et al. (1996); Giardino et al. (2003)]. PNS activity may be utilized to quantify stress by defining stress as a disruption of homeostasis with low PNS activity [Porges (1992)]. A state characterized by the absence of stress would therefore be one with high PNS activity [Porges (1992)]. The PNS activity can be quantified by measuring the RSA which manifests as excess power in the HRV power spectrum, at the respiratory frequency.

The connection between HRV and meditative states of mind has been well established in the scientific literature. Murata et al. (2004) collected EEG data and HRV data during Zen meditation, and analyzed the data in association with trait anxiety. It was found that slow alpha wave inter-hemispheric EEG coherence in the frontal lobe increased during meditation, reflecting non-task related cognitive processes such as attention. Among HRV measures, this was accompanied by an increase in the relative HF power and decrease in LF/HF, reflecting an increased parasympathetic response (the respiratory rate was fixed to 15 per minute, the high frequency band consists of frequencies between 9 min^−1^–24 min^−1^, the low frequency band consists of frequencies between 2.4 min^−1^–9 min^−1^). Wu and Lo (2008) reported HRV changes among two groups: the first group consisting of 10 experienced Zen practitioners, while the other group consisting of non-meditators. They found that when the ANS was under parasympathetic predominance, the heart rate can be purely modulated by respiration (their respiratory rate was about 15 per minute). Nesvold et al. (2012) studied HRV changes during non-directive meditation, and found an increase in both LF and HF components (the respiratory rate was unchanged, they interpret the change in HRV as entirely due to meditation, not changes in respiration). They also found no change in mean heart rate during meditation. Cysarz and Bussing (2005) investigated the impact of 4 exercises: spontaneous breathing, mental task, seated Zen, and walking meditation, on HRV. Seated Zen and walking meditation both resulted in a high degree of synchronization between respiration and heart rate, while spontaneous breathing and the mental task showed no such synchronization. The two kinds of meditation were characterized by increased LF (due to a much slower breathing rate) and in-phase RSA. Lo et al. (2019) studied the effect of Zen meditation on subjects undergoing a drug rehabilitation program, showing significant improvement in HRV (especially RSA), but no change in mean Heart Rate.

A popular HRV metric computed by many commercial wearable devices, and which is often regarded as a measure of the PNS is the Root Mean Squared value of the Successive Differences (RMSSD) of the interbeat intervals (henceforth “*RR* intervals”) [Natarajan et al. (2020)]. This is indeed the case when the HRV is measured during sleep, when the respiratory rate is typically in the range 11.8 min^−1^–19.2 min^−1^ [Natarajan et al. (2021)] and the RSA appears as excess power in the high frequency band of the HRV power spectrum. This is true because the RMSSD is a *biased* estimator of HRV, i.e. it preferentially weights high frequency components and is therefore, sensitive to RSA provided the respiratory rate is within the high frequency band. The RMSSD is not as informative about parasympathetic activity during slow paced breathing when the respiratory rate can be as low as 6 min^−1^ or even lower, and when the RSA falls within the low frequency band.

The goals of this article are twofold.

• To provide a pedagogical calculation based on Fourier analysis, to demonstrate that the RMSSD should only be considered as a marker of vagal or parasympathetic activity when the HRV is dominated by Fourier modes in the high frequency band, i.e. when the respiratory rate is ≳ 12 per min^−1^, which is commonly true during sleep.
• To propose an alternate metric to capture parasympathetic dominance ideally suited for periods of mindful breathing at low respiratory rates. We demonstrate the efficacy of this metric using a publicly available dataset of HRV measured during traditional meditation techniques.

When compared to an unbiased metric such as the Standard Deviation of the RR intervals (SDRR), we will see that the RMSSD greatly underestimates the HRV when the respiratory rate is low, i.e. during slow, paced breathing favored during mindful breathing meditation. The SDRR is however, a measure of the total ANS, and not the PNS. We will therefore consider another metric based on RSA called the *autonomic balance index* (ABI) which is the ratio of HRV due to respiration to the total HRV. When RSA is the dominant source of HRV, the *RR* interval time series resembles a periodic sine wave due to a small number of dominant Fourier components, a condition known as coherence [see for example, McCraty et al. (2009)]. Our computation of the ABI is qualitatively similar to the coherence ratio computation described in McCraty et al. (2009) (but the ABI is bounded between 0–1). We hypothesize that the ABI is proportional to the ratio PNS:ANS, and which can therefore be interpreted as a measure of the absence of stress. Proxies of autonomic balance have been considered in the literature, e.g. LF/HF or Poincare *S*_1_/*S*_2_, however, these measures do not work during slow, mindful breathing. During slow breathing, the RSA falls within the LF band, and HF power does not capture PNS activity, rendering the LF:HF ratio unsuitable. The Poincare *S*_1_ and *S*_2_ parameters are linearly related to RMSSD and SDRR and hence, they too cannot be used during slow breathing. We will provide a simple algorithm to compute the ABI. We will then apply this computation to a dataset of HRV measured during meditation, and show that the ABI is largest during mindful breathing.

## Methods

### Data

The data used for this analysis have been described by Peng et al. (1999) and may be downloaded from the Physionet database [Goldberger et al. (2000), https://physionet.org/content/ 120 meditation/1.0.0/]. Two specific meditative techniques were investigated by Peng et al. (1999): 1) Chinese Chi (Qigong) meditation and 2) Kundalini Yoga meditation. There were 8 Chi meditators (5 female and 3 male, age range 26–35, mean 29 years, with 1–3 months of prior practice) and 4 Kundalini Yoga meditators (2 female and 2 male, age range 20–52, mean 33 years, advanced meditators). Time series data of the instantaneous heart rate have been provided from which we computed the *RR* interval time series data. Data were collected during meditation and also during the period prior to meditation, which serves as a control. Also included were three additional non-meditating cohorts to serve as an additional control: 3) 14 healthy subjects (9 female, 5 male, age range 20–35, mean 25 years, supine) following metronomic breathing at 15 min^−1^, 4) 11 healthy subjects (8 female, 3 male, age range 20–35, mean 29 years) during sleep, and 5) 9 elite triathlon athletes (3 female, 6 male, age range 21–55, mean 39 years) during sleep. This is summarized in Table 1, ‘Duration’ refers to the average duration (minutes) of data per volunteer, and *N*_5_ is the total number of 5-min segments we used in the analysis.

**TABLE 1:** Seven cohorts of volunteers: Chi (med) and Yoga (med) are the two meditation cohorts, while Chi (rest) and Yoga (rest) are controls prior to meditation. Also considered are the metronomic breathing, normal healthy adults during sleep, and elite athletes during sleep. ‘Duration’ refers to the average time per volunteer (minutes), and *N*_5_ is the total number of 5-min segments we used in the analysis.

Cohort	Description	Volunteers	Duration (min.)	*N*_5_
Chi (med)	Chi meditation	8	57.1	67
Chi (rest)	Prior to meditation	8	58.3	54
Yoga (med)	Kundalini Yoga meditation	4	11.1	8
Yoga (rest)	Prior to meditation	4	10.1	6
Metronomic	Metronomic breathing	14	10.0	25
Sleep—Normal	Sleep—healthy individuals	11	352.6	535
Sleep—Ironman	Sleep—elite athletes	9	85.9	93

### Quantifying respiratory sinus arrhythmia

Let us now investigate a technique for quantifying the autonomic balance through RSA. There have been multiple methods suggested for quantifying RSA [see for e.g. Grossman et al. (1990)]. In this article, we consider a different technique similar to well known HRV metrics, and one which is suitable for use during slow, paced breathing, especially when the condition of coherence is attained. McCraty et al. (2009) quantify the condition of coherence through the coherence ratio. Here we will consider a similar approach: We first compute the standard deviation of the RR intervals due to RSA alone, called the SDRSA, considered to be a probe of the PNS. We then compute the ratio (SDRSA/SDRR) which is bounded between 0–1, and which we refer to as the Autonomic Balance Index (ABI).

A required step in computing the SDRSA is the measurement of the respiratory rate. A possible complication here is that the respiratory rate is variable when the subject is awake. We therefore consider segments that are short enough that we may make the assumption that the respiratory rate is approximately constant within that segment. It is also important that the chosen segment is not too short because 1) a very short time window will admit only a small number of realizations of each Fourier mode, increasing the shot noise error, and 2) the resolution in the spectral domain is inversely proportional to the size of the window in the time domain. We choose a segment size of 2 min and smooth the signal with a Hann window. Estimation of SDRSA and ABI follow the algorithm.

1 Define 2 frequencies *f*_1_ and *f*_2_ that may be considered the lower and upper bounds for the respiratory rate within each 2 min segment.
2 Compute the power spectral density (PSD) normalized so that *∫df P*(*f*) = SDRR^2^. The PSD is interpolated using a cubic spline. Let *f*_0_ be the frequency that corresponds to the peak of the PSD, and let *A*_0_ be the peak value.
3 Fit a Gaussian $G\left(f\right)=A_{0}\text{exp}-\frac{1}{2}{\left(\frac{f-f_{0}}{\sigma }\right)}^{2}$ to the peak of the PSD described by a mean value (*f*_0_), a standard deviation (*σ*), and amplitude *A*_0_.
4 Construct the residual *R*(*f*) = *PSD*(*f*) − *G*(*f*). From the residual, identify the largest peak amplitude *A*_1_ in the range *f*_1_ < *f* < *f*_2_. Compute the ratio *P* = *A*_0_/*A*_1_ which represents the prominence of the main peak *A*_0_. If *P* is greater than a preset limit *P*_min_, it validates our assumption that the PSD is dominated by a single respiratory frequency. If *P* < *P*_min_, no values are returned and the data are discarded.
5 If *P* > *P*_min_, we compute the following two quantities: 1) The variance due to respiration = SDRSA^2^, estimated by the area under the Gaussian curve = $\sqrt{2\pi }\;A_{0}\;\sigma$ . 2) The normalized quantity ABI = $\left[SDRSA/SDRR\right]$ . The algorithm returns the estimated respiratory rate *f*_0_ and ABI.

We apply the algorithm above during meditation, metronomic breathing, and sleep. For the data measured during rest prior to meditation (Chi (rest) and Yoga (rest)) however, we include an additional step at the beginning because the respiratory rate is highly variable, and the algorithm works best when the respiratory range is fairly small. We compute the frequency *f*_0_ corresponding to the peak of the PSD and set *f*_1_ = MAX (12 min^−1^, *f*_0_−3 min^−1^) and *f*_2_ = MIN(22 min^−1^, *f*_0_+3 min^−1^). For the meditation cohorts (Chi (med) and Yoga (med)), we set *f*_1_ = 3 min^−1^ and *f*_2_ = 10 min^−1^. For the metronomic, normal, and ironman cohorts, we set *f*_1_ = 10 min^−1^ and *f*_2_ = 20 min^−1^. In all cases, we set *P*_min_ = 2.

The data are initially divided into non-overlapping 5 min segments. Each 5 min segment is then divided into a number of 2 min segments with an overlap of 10 s. To ensure sufficient data for analysis, we estimate the coverage in each 2 min segment as the number of observed heart beats/expected number of heart beats, and impose the condition that the coverage > .7. Provided the coverage condition is met, *f*_0_ and ABI are estimated from each such 2 min segment (starting from 2:00, in increments of 10 s, until the 5:00 min mark). A total of 19 such estimates can be made from a 5 min segment of data (from 2:00 to 5:00 in increments of 10 s, including both endpoints). The median value of these different estimates is then calculated for *f*_0_ and ABI provided there is a minimum of 9 estimates. If there are fewer than 9 estimates (for example, due to missing data), we do not store any results for that 5 min segment.

## Results

### An analytic approximation for the root mean square of successive differences and standard deviation of RR intervals

As mentioned in the Introduction, the RMSSD is influenced by the respiratory rate and is therefore hard to interpret. Here, we provide a pedagogical approximation of the RMSSD and SDRR from first principles, using example data measured during slow breathing.

Let *RR*_*i*_ represent the *i*th value of the *RR* interval time series sampled at time intervals *t*_*i*_ = [*t*_1_, *t*_2_, *t*_3_, ⋯ ], where *RR*_*i*_ = *t*_*i*_−*t*_*i*−1_. The *RR* time series contains a constant term ⟨*RR*⟩ and a fluctuating term $\tilde{RR}$ :

$$RR_{i}=\left\langle RR\right\rangle +{\tilde{RR}}_{i}.$$ (1)

The standard deviation of the RR intervals (SDRR) is computed as:

$$SDRR={\left\langle {\tilde{RR}}_{i}^{2}\right\rangle }^{1/2},$$ (2)

where the angle brackets represent the mean value. The fluctuating component is expected to be much smaller than the mean, i.e. SDRR ≪⟨*RR*⟩. The differences between successive *RR* intervals Δ*RR*_*i*_ may be computed as:

$$\begin{array}{cc} \Delta RR_{i} & =RR_{i}-RR_{i-1}\\ & =\frac{RR_{i}-RR_{i-1}}{\Delta t_{i}}\Delta t_{i}\\ & \approx \langle RR\rangle \;\frac{d\tilde{RR_{i}}}{dt}\;\left[1+\frac{\tilde{RR_{i}}}{\langle RR\rangle }\right]\\ & \approx \langle RR\rangle \;\frac{d\tilde{RR_{i}}}{dt}\\ & \approx \frac{60\;bpm}{\langle HR\rangle }\;\frac{d\tilde{RR_{i}}}{dt}, \end{array}$$ (3)

where we used Δ*t*_*i*_ = *t*_*i*_−*t*_*i*−1_ = *RR*_*i*_, and we ignored term of quadratic order in $\tilde{RR_{i}}$ . ⟨*HR*⟩ is the mean heart rate, and “bpm” stands for beats per minute. The RMSSD is the root mean square of the successive differences Δ*RR*_*i*_, i.e.

$$RMSSD={\left\langle {\left(\Delta RR_{i}\right)}^{2}\right\rangle }^{1/2}.$$ (4)

Let us interpolate and re-sample the ${\tilde{RR}}_{i}$ sequence at a sampling frequency *N*/*T*_0_ to obtain an $\tilde{RR}\left(t\right)$ field with *N* samples (and where *T*_0_ is the length of the signal under consideration, we will assume that the end points are identified to mimic periodicity). We may expand this in a Fourier series:

$$\begin{array}{cc} {\tilde{RR}}_{i} & \approx \overset{n_{\text{F}}}{\underset{n=1}{\sum }}a_{n}\;\text{cos}\frac{2\pi n}{T_{0}}t_{i}+b_{n}\;\text{sin}\frac{2\pi n}{T_{0}}t_{i}\\ & \approx \overset{n_{\text{F}}}{\underset{n=1}{\sum }}w_{n}\;\text{sin}\left[\frac{2\pi n}{T_{0}}t_{i}+\varphi_{n}\right], \end{array}$$ (5)

where *n*_F_ ≤ *n*_max_ is the total number of Fourier modes to be included in the approximation, and by Nyquist’s theorem, *n*_max_ = *N*/2. $w_{n}=\sqrt{a_{n}^{2}+b_{n}^{2}}$ , $\varphi_{n}={\text{tan}}^{-1}\left(\frac{a_{n}}{b_{n}}\right)$ , and *t*_*i*_ are the time intervals at which the *RR* intervals are calculated. We have assumed *b*_*n*_ ≠ 0 and $-\frac{\pi }{2}<{\text{tan}}^{-1}\left(\frac{a_{n}}{b_{n}}\right)<\frac{\pi }{2}$ . The coefficients *a*_*n*_ and *b*_*n*_ may be computed as:

$$\begin{array}{cc} a_{n} & =\frac{2}{T_{0}}\int_{0}^{T_{0}}dt\;\tilde{RR}\left(t\right)\;\text{cos}\frac{2\pi nt}{T_{0}}\\ b_{n} & =\frac{2}{T_{0}}\int_{0}^{T_{0}}dt\;\tilde{RR}\left(t\right)\;\text{sin}\frac{2\pi nt}{T_{0}}. \end{array}$$ (6)

The frequencies of the Fourier modes are $f_{n}=\frac{n}{T_{0}}$ for *n* = 1, 2, 3, ⋯*n*_F_. Let SDRR (*n*_F_) and RMSSD (*n*_F_) be the values of SDRR and RMSSD estimated using *n*_F_ ≤ *n*_max_ Fourier modes, while SDRR and RMSSD are the exact values. Eq. 5 can be used to estimate SDRR (*n*_F_) from Eq. 2. From Eqs. 3, 5, we find

$$\Delta RR_{i}\left(n_{\text{F}}\right)\approx \frac{60\;bpm}{\left\langle HR\right\rangle }\;\frac{2\pi }{T_{0}}\;\overset{n_{\text{F}}}{\underset{i=1}{\sum }}n\;\omega_{n}\;\text{cos}\left(\frac{2\pi n}{T_{0}}\;t_{i}+\varphi_{n}\right),$$ (7)

from which we can estimate the RMSSD (*n*_F_) using Eq. 4.

**FIGURE 1:**
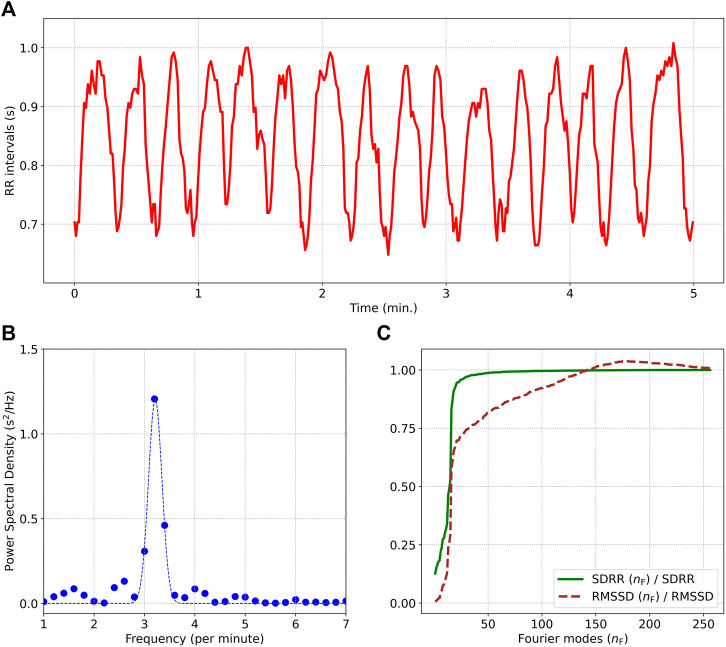
**(A)** describes a 5 min segment of the *RR* interval time series, from a subject practicing Chi meditation. **(B)** Shows the power spectral density showing strong respiratory sinus arrhythmia at a respiratory frequency of 3.2 min^−1^. The ratios (RMSSD (*n*_F_)/RMSSD) and (SDRR (*n*_F_)/SDRR) computed from the first *n*_F_ Fourier modes are displayed in **(C)**. Many more Fourier modes need to be included for the computation of RMSSD (*n*_F_) compared to SDRR (*n*_F_) since RMSSD preferentially weights the higher frequency modes.

Figure 1A shows a 5 min sample of RR intervals from a participant practicing Chi meditation, exhibiting high HRV and prominent RSA. Plot b) shows the power spectral density along with the best fit Gaussian curve. We note that most of the power comes from a narrow band of frequencies centered around a respiratory frequency of 3.2 min^−1^. Plot c) shows the ratios [RMSSD (*n*_F_)/RMSSD] and [SDRR (*n*_F_)/SDRR], where the values of RMSSD (*n*_F_) and SDRR (*n*_F_) are calculated from the approximate formula we derived, and include up to *n*_F_ Fourier modes. RMSSD and SDRR are the exact values. Both RMSSD (*n*_F_) and SDRR (*n*_F_) approach their true values as *n*_F_ → *n*_max_, but the RMSSD (*n*_F_) computation made some simplifying assumptions, and is hence not as accurate as the SDRR (*n*_F_) computation. RMSSD (*n*_F_) also converges to the true value with a far larger number of Fourier modes than SDRR (*n*_F_) since it preferentially weights high frequency modes. SDRR (*n*_F_) requires only 18 Fourier modes (corresponding to a peak frequency of 88/5 = 3.6 min^−1^) to reach 90% of the true value. This makes intuitive sense since the peak of the PSD was found to be at 3.2 min^−1^ and there is very little power at higher frequencies. RMSSD (*n*_F_) on the other hand, requires 88 Fourier modes (corresponding to a peak frequency of 88/5 = 17.6 min^−1^) to reach 90% of the true value. Frequencies above 3.6 min^−1^ contribute ≲ 10% to the SDRR, while contributing ≈35% to the RMSSD. This discussion highlights a major flaw in using the RMSSD to quantify HRV for slow breathing: A small amount of power at high frequencies is preferentially weighted by the RMSSD even though the high frequency Fourier modes in this case are not associated with respiration. The SDRR being an unbiased HRV estimator does not weight Fourier modes differently by frequency. The lack of high frequency content also results in the RMSSD being much lower than the SDRR. For this example, we find RMSSD = 36.8 ms, while the SDRR = 102.6 ms.

Figure 1 demonstrates a sample wherein most of the variance is contributed by a single Fourier mode (or a narrow range of modes). Let us consider the special case when all the power comes from a single frequency mode. The *RR* time series may then be simplified as:

$$RR=A\;\text{sin}\left[2\pi ft+\varphi \right],$$ (8)

where *f* is the respiratory rate and *A* is the amplitude of oscillations. The SDRR is then simply $A/\sqrt{2}$ . We can approximate Δ*RR* as:

$$\begin{array}{cc} \Delta RR & \approx \Delta T\frac{d\left(RR\right)}{dt}\\ & \approx \frac{60\;bpm}{\langle HR\rangle }\;\frac{d\left(RR\right)}{dt}\approx \frac{2\pi \;f}{\langle HR\rangle }\;A\;\text{cos}\left(2\pi ft+\varphi \right), \end{array}$$ (9)

where *f* is measured in min^−1^ and ⟨*HR*⟩ is measured in beats per minute. Taking the root mean square of Eq. 9, we get:

$$RMSSD\approx 1.05\;SDRR\;\left(\frac{72\;bpm}{\left\langle HR\right\rangle }\right)\;\left(\frac{f}{12\;{min}^{-1}}\right).$$ (10)

It is clear from Eq. 10 that the RMSSD increases with respiratory rate. This metric is therefore best employed in situations when the respiratory rate is high (i.e. > 12 min^−1^) and relatively constant, i.e. during sleep. In the example shown in Figure 1, we find the respiratory rate = 3.2 min^−1^, and the mean heart rate is 72 beats per minute. From our approximate analysis (Eq. 10), we expect a ratio RMSSD:SDRR = .28, while the true ratio = .36.

### Autonomic balance during meditation

We have seen in the previous subsection that the RMSSD is unsuitable during periods of slow, paced breathing favored by mindful breathing meditation. The SDRR, although an unbiased metric, is also not ideal for biofeedback during mindful breathing meditation since it captures the total variance, i.e. it is a measure of the ANS and not the PNS. In this subsection, we apply the algorithm for computing ABI described in the Methods section, to the data.

**FIGURE 2:**
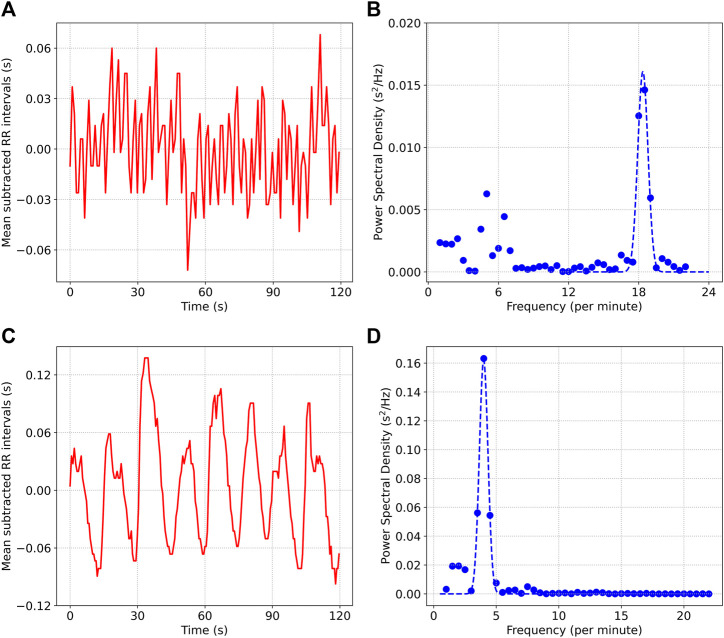
**(A)** and **(B)** show the mean subtracted RR interval time series data and the power spectral density, from a 2 min segment of data collected prior to meditation. **(C)** and **(D)** show the same quantities for data collected during Kundalini Yoga meditation.

Figure 2 shows the mean subtracted RR interval time series data for two situations: a) describes a 2 min resting period prior to meditation, while c) shows the data during meditation (we used an sample from the Kundalini Yoga cohort for this figure). b) and d) show the power spectral density plots for the two situations respectively. Contrasting the two scenarios, we note the following: 1) The respiratory rate is much lower during the meditation phase (4 min^−1^) compared to the resting phase (18.5 min^−1^), 2) Most of the power is contained within the respiratory band during the meditation phase (ABI = .86). In the case of the resting period prior to meditation, there is considerable power at frequencies not associated with respiration {ABI = .57, some of this power is likely due to Mayer Wave Sinus Arrhythmia [Julien (2006; 2020)]}, 3) The amplitude of oscillations is much larger during meditation (SDRR = 55.7 m) compared to the resting phase (SDRR = 26.4 m).

**FIGURE 3:**
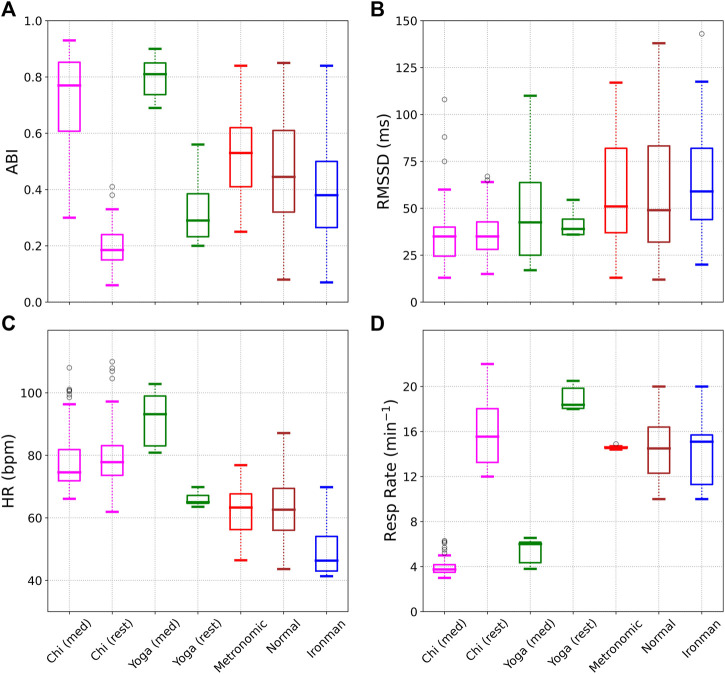
A comparison of the autonomic balance index (ABI), RMSSD, Heart Rate, and Respiratory Rate for the 7 different cohorts. ABI shows a very significant difference between the meditation and rest data, while such a difference is not seen in the RMSSD. The respiratory rate is also substantially decreased during meditation compared to the rest phase.

Figure 3 shows various metrics evaluated for the 7 different cohorts: 1) Chi (med), i.e. during Chi meditation, 2) Chi (rest), i.e. prior to meditation, 3) Yoga (med) during Kundalini Yoga meditation, 4) Yoga (rest) prior to meditation, 5) Metronomic breathing, 6) Normal, i.e. healthy individuals during sleep, and 7) Ironman triathletes during sleep. The Chi cohort (meditation and rest) is shown in magenta, the Yoga cohort (meditation and rest) in green, metronomic breathing in red, normal in brown, and ironman in blue. Subplot a) shows the ABI for the 7 cohorts. The highest scores are obtained for the two meditation cohorts, followed by metronomic breathing, sleep, and finally the rest cohort (awake, but not meditating). Subplot b) shows the RMSSD. Not surprisingly the meditation cohorts perform poorly in the RMSSD comparison due to the dependence of RMSSD on respiratory rate. Subplot c) shows the average heart rate. There is very little difference in heart rate during Chi meditation. For the Kundalini Yoga cohort however, the heart rate *increases* during meditation. The heart rate is lowest during sleep, especially for the elite athletes. We note the heart rate is not expected to decrease significantly during mindful breathing [Shaffer and Ginsberg (2017)], and is therefore not a good metric to use as biofeedback. Subplot d) shows the respiratory rate. We see that the two meditation techniques we discuss here encourage very slow breathing. As expected the metronomic breathing cohort shows very little variability.

Table 2 shows the mean (standard deviation) computed from the 5-min medians, for the autonomic balance index (ABI), respiratory rate, RMSSD, SDRR, heart rate (HR), LF power, and HF power for the seven different cohorts. One can compare a pair of cohorts by means of the Cohen *d* effect size [Cohen (1992); Coe (2002)]:

$$d=\frac{\mu_{2}-\mu_{1}}{\sigma },$$ (11)

where *μ*_1_ and *μ*_2_ are the means of the two cohorts, and *σ* is the pooled standard deviation given by:

$$\sigma^{2}=\frac{\sigma_{1}^{2}\left(N_{1}-1\right)+\sigma_{2}^{2}\left(N_{2}-1\right)}{N1+N2-2}.$$ (12)

One can also define a *Z*−score

$$Z=\frac{\mu_{2}-\mu_{1}}{\sigma_{z}},$$ (13)

from which one may obtain a *p*−value to determine statistical significance. *σ*_*z*_ is defined as

$$\sigma_{z}^{2}=\frac{\sigma_{1}^{2}}{N_{1}}+\frac{\sigma_{2}^{2}}{N_{2}}.$$ (14)

*N*_1_ and *N*_2_ are the number of samples in the two cohorts.

**TABLE 2:** Mean (standard deviation) computed for the 7 cohorts, for different metrics.

Activity	ABI	Resp. Rate	RMSSD	SDRR	HR	LF	HF
		(min^−1^)	(ms)	(ms)	(bpm)	(ms^2^)	(ms^2^)
Chi (med)	.72 (.17)	3.9 (.8)	35.5 (16.3)	66.9 (22.5)	77.8 (9.7)	3,365 (2,700)	312 (298)
Chi (rest)	.19 (.07)	15.8 (2.8)	36.4 (12.8)	56.8 (14.7)	79.7 (10.1)	1,167 (790)	391 (217)
Yoga (med)	.80 (.07)	5.4 (1.1)	50.4 (31)	86.6 (32.3)	91.9 (8.5)	6,574 (4,746)	644 (776)
Yoga (rest)	.33 (.12)	18.9 (1.1)	41.6 (6.7)	56.8 (16)	66 (2.2)	1,312 (690)	470 (109)
Metron	.52 (.15)	14.6 (.1)	59.1 (30.2)	58.1 (20.6)	61.7 (8.1)	828 (572)	1,618 (1,382)
Normal	.45 (.18)	14.6 (2.7)	58.1 (32.2)	67.9 (39.8)	62.1 (9.1)	1,676 (1990)	1728 (1994)
Ironman	.40 (.18)	14.1 (2.4)	63.5 (25.4)	78.8 (46.5)	49.4 (7.1)	2,176 (4,128)	1933 (3,289)

Table 3 shows the effect size and fractional change, when comparing two cohorts: The rest cohort (pre-meditation) and the meditation cohort, for both Chi, and the Yoga meditation. The Cohen’s *d*−effect size compares rest to meditation, so that when the effect size is positive, the metric measured for the meditation group is larger than the same metric measured for the rest group. The fractional change is defined as: [ metric (med) - metric (rest) ]/metric (rest). Thus, a positive fractional change implies that the metric is higher in the meditation group compared to the rest group. The column for statistical significance tests whether the difference is statistically significant at the *p* < .05 level for a 2-tailed test (we used the condition |*Z*| > 1.96). The sample sizes are provided in Table I.

**TABLE 3:** Comparison of the rest and meditation cohorts. When the effect size and fractional change are positive, the metric measured for the meditation cohort is larger. A negative effect size and fractional change imply that the metric measured for the rest cohort is larger.

Metric	Chi meditation			Yoga meditation		
	Effect Size	Stat. Sig. ?	Fractional Change	Effect Size	Stat. Sig. ?	Fractional Change
ABI	3.9	Yes	2.79	5.0	Yes	1.42
Resp. Rate	−6.1	Yes	−.75	−12.3	Yes	−.71
RMSSD	−0.1	No	−.03	0.4	No	.21
SDRR	0.5	Yes	.18	1.1	Yes	.53
LF	1.1	Yes	1.88	1.4	Yes	4.01
HF	−0.3	No	−.20	0.3	No	.37
HR	−0.2	No	−.02	3.9	Yes	.39

The metrics that show the largest difference between rest and meditative states are the respiratory rate and the ABI. Not surprisingly, the respiratory rate decreases during meditation. The Cohen’s *d*−effect sizes for the respiratory rate are *d* = −6.1 for Chi, and *d* = −12.3 for Yoga. The ABI shows a large positive effect size (*d* = 3.9 for Chi and *d* = 5.0 for Yoga). Among the other metrics we considered, only the SDRR and LF power are statistically significant for both forms of meditation. For SDRR, we find *d* = .5 (1.1) for Chi (Yoga), while for LF power, we find *d* = 1.1 (1.4) for Chi (Yoga). The HR is statistically significant for the Yoga cohort, but not for the Chi cohort. Nevertheless, we do not recommend using HR as a biosignal since it *increased* during meditation (Yoga cohort) in this particular example. The cohort with the lowest heart rate was “Ironman”, i.e. the elite athletes. We note that the effect size computation for the Yoga (rest) and Yoga (med) cohorts needs to be treated with caution, due to the small sample sizes.

### Real time biofeedback

We have shown that the ABI is greatly increased during mindful breathing. In this subsection, we discuss how the ABI can potentially be used as a biofeedback tool during meditation, or else, to monitor stress [for a systematic review of biofeedback and stress management, see for e.g. Yu et al. (2018)].

HRV biofeedback is often implemented by encouraging slow, paced breathing at the resonance frequency, resulting in heart rate oscillations that are much larger than baseline values [Shaffer and Meehan (2020)]. HRV biofeedback has been shown to be a promising intervention for reducing anxiety and stress [Goessl et al. (2017)]. In laboratory tests, HRV biofeedback has been shown to improve cognitive performance, while aiding relaxation [Prinsloo et al. (2011)]. HRV biofeedback may be effectively gamified, i.e. presented in a visually compelling manner that encourages users to engage in activities that lower stress [Yu et al. (2017)]. It has also shown promise in the treatment of trauma and post-traumatic stress disorder [Reyes (2014)]. Studies have indicated that RSA biofeedback appears to be more effective in reducing state anxiety and heart rate stress reactivity compared to passive relaxation techniques [Sherlin et al. (2009)].

**FIGURE 4:**
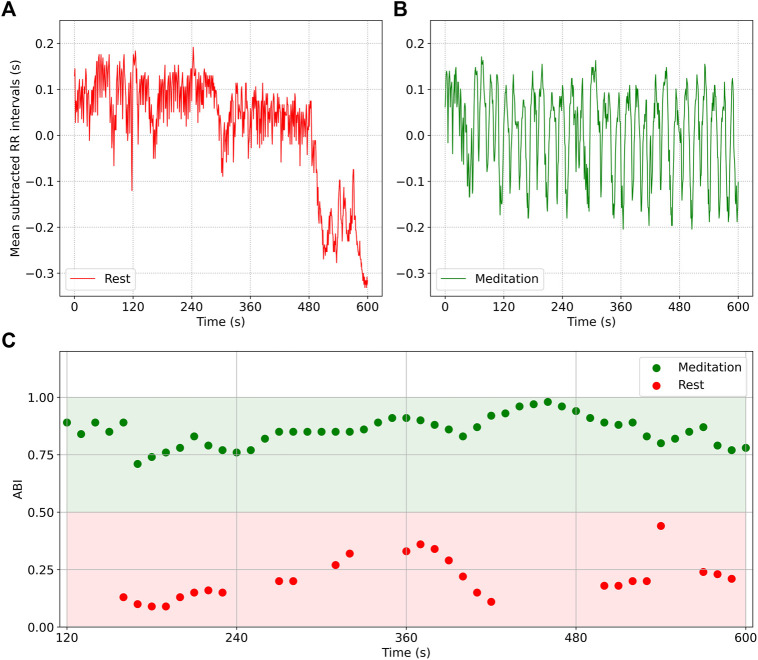
RR interval data (mean subtracted) from a participant at rest **(A)** and during Chi meditation **(B)**. RSA is noticeably present in both plots, but is dominant in **(B)**. Plot **(C)** shows the ABI computed from 2 min of data. The red data points are plotted for the waveform in **(A)**, while the green data points are plotted for the waveform in **(B)**. The ABI = .5 line is an approximate boundary that separates focused meditation from other activities.

Figure 4 shows how the ABI may be used to implement a real time biofeedback mechanism, providing feedback every 10 s. Plot a) shows a 10 min segment of RR interval (mean subtracted) data from a participant in the Chi (rest) cohort, i.e. during rest, while b) shows a 10 min segment from the same participant in the Chi (med) cohort, i.e. during meditation. While sinus arrhythmia is noticeable in both plots, it is far more dominant in b) than in a). Plot c) shows the ABI measured in 2 min windows, and updated every 10 s: The red data points are plotted for the waveform shown in a) when the participant was at rest, while the green data points are computed for the waveform in b), i.e. during meditation. A rough guide that distinguishes meditation from ordinary daily activities could be ABI = .5, i.e. when the ABI > .5, the RSA component contributes more than half of the HRV, indicating a dominance of the PNS.

Two important design criteria regarding biofeedback are: 1) Window size used for computing the ABI and respiratory rate, and 2) How often the ABI and respiratory rate are computed. In this example, we computed the ABI using 2 min of data, but updated the computation every 10 s. The rationale for choosing 2 min for the window size was discussed in the Methods section. Since the time between measurements is far smaller than the measurement window, successive readings are highly correlated. Nevertheless, a trend is noticeable: in some cases, the measurements show a steady increase, indicating an increasing contribution due to RSA, while in other cases, there is a steady decrease, possibly indicating stress or a momentary distraction. A simple algorithm can then use the instantaneous value of ABI along with the trend to provide corrective feedback. In this way, HRV biofeedback could be used to train concentration and mindfulness of breathing. It is also noticeable that there are gaps in the plot shown in c), which could be due to a number of reasons. Noisy data due to motion will impair our ability to measure ABI. In some cases RSA is not noticeable or too weak for detection. In other cases, the respiratory rate may be too variable for a measurement to be made with high confidence. In these situations, the gaps may be filled though time series forecasting using data from prior measurements.

## Discussion

In this article, we discussed heart rate variability measured during mindful breathing meditation. We first considered the RMSSD and SDRR, two popular HRV metrics used by commercial wearable devices to quantify HRV. We derived an approximate but pedagogical, analytic expression for SDRR (*n*_F_) and RMSSD (*n*_F_) using Fourier decomposition, and including the first *n*_F_ number of Fourier modes. This pedagogical exposition made it clear that the RMSSD is a *biased estimator* of the HRV in that it preferentially weights higher frequency Fourier components, with the result that a small amount of power at high frequencies can contribute a disproportionately large influence on the RMSSD. Such an effect is not seen in the SDRR which weights all Fourier modes equally. RMSSD is thus, not a suitable metric to quantify HRV during slow, mindful breathing.

We have suggested a metric that quantifies the fraction of HRV contributed by the RSA as a HRV metric that is ideally suited to serve as a biofeedback signal during mindful breathing meditation. The ABI metric was motivated by the spectral properties of HRV during mindful breathing, and is qualitatively similar to the coherence ratio computation described in McCraty et al. (2009). During mindful breathing, most of the power falls within the respiratory band of frequencies, with very little power at lower frequencies (note that the respiratory frequency itself may be as low as ∼ 3 min^−1^) indicating that most of the HRV is due to respiratory sinus arrhythmia. Unlike other HRV measures, ABI is less influenced by age, gender, physical fitness etc as it is a ratio of two HRV measures. Instead, it is most influenced by practices that result in PNS dominance, e.g. meditation. We described a simple algorithm to compute ABI from the power spectral density of *RR* fluctuations.

We then applied the ABI to heart rate time series data collected during meditation, and described in Peng et al. (1999), who found extremely prominent HRV fluctuations during two specific, traditional meditation techniques: Chinese Chi and Kundalini Yoga (here denoted as Chi (med) and Yoga (med) respectively). The data also included a period of rest prior to meditation (here denoted as Chi (rest) and Yoga (rest) respectively). As additional controls, the data also included metronomic breathing (“metronomic”), healthy adults during sleep (“normal”), and elite athletes during sleep (“ironman”). The values of ABI and RMSSD for the 7 different cohorts are shown in Figure 3A; Figure 3B and demonstrate that ABI is a more sensitive metric than the RMSSD. The mean and standard deviation of ABI, Respiratory Rate, RMSSD, SDRR, heart rate (HR), LF power, and HF power were tabulated for the 7 cohorts in Table 2, while Table 3 listed the effect size and fractional change for various metrics comparing the rest and meditation cohorts for Chi and Yoga forms of meditation. The respiratory rate showed the largest (negative) effect size (*d* = −6.1 for Chi and *d* = −12.3 for Yoga) indicating a very significant decrease in the respiratory rate. The ABI showed a large (positive) effect size (*d* = 3.9 for Chi and *d* = 5.0 for Yoga) indicating that the ABI increases significantly. Among the other metrics, only the SDRR [*d* = .5 (1.1) for Chi (Yoga)) and LF power (*d* = 1.1 (1.4) for Chi (Yoga)] showed a statistically significant change for both types of meditation.

We do not recommend the use of the LF/HF metric to measure autonomic balance during slow breathing. The LF/HF metric is most useful during sleep when the respiratory rate is typically high enough for vagal activity to manifest in the HF band of the HRV power spectrum. In that case, the LF/HF ratio indeed has useful information, and values of *LF*/*HF* ≲ 1 indicate parasympathetic dominance. From Table 2, we note that the two cohorts “Normal” and “Ironman”, i.e. healthy adults and elite athletes showed a mean LF/mean HF ratio close to 1.0 during sleep. The cohort labeled “Metron”, i.e. metronomic breathing at ≈ 15 per minute had the lowest value of mean LF/mean HF = .5 indicating PNS dominance. This reasoning however does not apply to the two meditation cohorts: The very low respiratory rates seen in these two cohorts causes vagal activity to appear in the LF band instead of the HF band. When the breathing rate is lower than 9 min^−1^, the HF power should not be interpreted as indicative of PNS activity and the LF/HF ratio should not be considered for situations involving slow breathing. From Table 2, we see that HF ≪ LF for the two meditation cohorts, and since the RSA is entirely contained within the LF band, the HF power is not of any real physiological significance. The failure of the mean LF:HF ratio to signify autonomic balance underscores the need for a different metric that does not depend on respiratory rate. The ABI provides such a metric.

There are several limitations to this work. The ABI algorithm interprets power at frequencies outside the chosen range (*f*_1_, *f*_2_) of possible respiratory rates as due to stress, i.e. sympathetic nervous system activity creates low frequency power. However not all power at low frequencies is due to stress, e.g. the Mayer Wave Sinus Arrhythmia (MWSA) at *f* ≈ 6 per minute is caused by blood pressure oscillations [Julien (2006; 2020)], and a pronounced MWSA can cause an artificially low ABI when the RSA occurs at much higher frequencies. Another limitation in computing the ABI is that it assumes a constant respiratory rate within a 2 min window. While this is naturally satisfied during mindful breathing or during sleep, that is less likely to be the case when subjects are awake. We have also assumed that the inhalation and exhalation times are the same. ABI also relies on the presence of respiratory sinus arrhythmia. We were able to compute ABI in the period prior to meditation, but we expect it would be harder to do so at a random time of day, and especially during times of stress when the respiratory sinus arrhythmia would be subdominant. RSA is also decreased in older individuals and it would therefore be harder to compute ABI for older subjects. The algorithm is unlikely to work in individuals who have heart arrhythmias. ABI would likely not be computed during normal activities such as working, eating, watching television, etc, and other HRV metrics would be preferable during these activities. Wearable devices also have difficulty measuring *RR* interval data when subjects are moving.

## Data Availability

The original contributions presented in the study are included in the article/supplementary material, further inquiries can be directed to the corresponding author.
